# Supplementary material for: Abuse recognition by shelter staff and shelter animal adopters
Source: PLoS One. 2026 Mar 11;21(3):e0343066. doi: 10.1371/journal.pone.0343066 (PMC12978493; doi:10.1371/journal.pone.0343066)
Supplement: S1 File — (DOCX) [file pone.0343066.s001.docx]

**Supplement 1 – Shelter staff and adopter questions**

**Introduction**

Please regard all questions on animal abuse in light of animal physical abuse: a wide range of injurious acts requiring active engagement (Arkow, 2015, p. 352) such as beating, kicking, throwing, shaking, possibly using an object. Although many other forms of animal abuse exist, this survey focuses on these forms of physical harm (which may come with mental harm in addition). There are no right or wrong answers, please fill in the first answer that comes to mind.

Reference: Arkow P. Recognizing and responding to cases of suspected animal cruelty, abuse, and neglect: what the veterinarian needs to know. Veterinary Medicine: Research and Reports. 2015 Nov 5:349-59.

**Questions on abuse**

Based on an animal’s behaviour, do you think you can identify whether it is being mistreated by its owner? Yes, in most cases, Yes, sometimes, No, Don’t know/unsure.

Which of these signs would you regard as possible indicative of an animal’s historic abuse experience? Very unlikely, Somewhat unlikely, Neutral, Somewhat likely, Very likely.

An animal is wary of adult contact.

An animal seems frightened by owner(s) or of going towards them.

An animal seems frightened by going home.

An animal seems less capable of forming a social bond with humans.

An animal acts apprehensive when a human (adult or child) cries.

An animal uses demanding (manipulative) behaviour to get attention.

An animal displays a vacant or frozen gaze.

An animal displays behavioural extremes, such as aggressiveness.

An animal displays behavioural extremes, such as withdrawal.

An animal sits or lies very still while surveying surroundings.

A young animal shows inappropriate or precocious maturity.

How would you rate the accuracy of below animal behavioural signs as indicative of an animal’s abuse experience? 7-point Likert-scale (1 = Very inaccurate; 7 = Very accurate).

withdrawal

anxiety

depression

auto mutilation

aggression

behavioural problems

hypervigilant, guarded/insecure behaviour

anxiety or reluctance to be in the presence of or be left alone with a particular person or to go to a particular place/room, behavioural avoidance

acting overly 'pleasing' through for instance submissive behaviour/appeasement

expressing person specific fear

sudden behavioural change, which cannot be explained otherwise

problems with forming a social bond with humans

expressing person specific overly 'pleasing' behaviour through for instance submissive behaviour/appeasement

**Questions on background**

Shelter staff

Finally, would you be willing to provide us additional information regarding your back ground?

Which description most accurately describes your present job? Animal caretaker – dog, Animal caretaker – cat, Animal caretaker – cat and dog, Animal trainer, Animal behavioural therapist, Paravet, Paravet and animal behavioural therapist, Paravet and animal caretaker – dog, Paravet and animal caretaker – cat, Paravet and animal caretaker cat and dog, Vet, Other.

How many years of experience do you have in your present job? 0-2, 2-5, 5-10, 10-15, >15.

How many years of experience do you have in other job(s) in the animal shelter environment (so in addition to the years mentioned in the before question)? 0-2, 2-5, 5-10, 10-15, >15.

Did you finalise an education regarding animals? No, Yes, animal caretaker, yes, animal shelter management, yes, para veterinary, yes, veterinary, yes, trainer or instructor, yes, cat behavioural therapist, yes, dog behavioural therapist, other.

With which gender do you identify? Male, Non-binary, Female, Other, Prefer not to say.

Which is your age category? 18-35, 35-50, 50-65, >65 year, Prefer not to say.

At the moment, do you have one or more: Dog(s), Cat(s), Other animal(s).

Historically, did you have one or more: Dog(s), Cat(s), Other animal(s).

Do you know approximately how many of the below animals enter the rehoming organisation where you work on a yearly basis? Dogs, Cats.

With some of these animals, is information provided that animal abuse has taken place, by purposely hurting an animal through for example hitting, stomping, kicking, shaking? Yes, by an owner, Yes, by an official such as of local government, police or an inspection body, Yes, by another organisation. No, that does not occur in our rehoming organisation.

Shelter adopters

Which animal did you adopt? Cat/dog.

Did the rehoming organisation provide information on the animal’s historic abuse? Yes, indicated with certainty, Yes, indicated with likeliness, No, not known, Other.

Do you think the animal was abused before it came to live with you? Yes, with certainty, Yes, likely, No.

If you think the animal was mistreated before it came to live with you, how? Neglect, such as too little food provided, mentally abused, such as scared or isolated, physically abused, such as through kicking or hitting, other.

With which gender do you identify? Male, Non-binary, Female, Other, Prefer not to say.

Which is your age category? 18-35, 35-50, 50-65, >65 year, Prefer not to say.
