# Supplementary material for: Abuse recognition by shelter staff and shelter animal adopters
Source: PLoS One. 2026 Mar 11;21(3):e0343066. doi: 10.1371/journal.pone.0343066 (PMC12978493; doi:10.1371/journal.pone.0343066)
Supplement: S2 File — (DOCX) [file pone.0343066.s002.docx]

**Supplement 2 – Behavioural signs from child-directed studies on child abuse recognition**

Behavioural signs selected from three literature sources, which we provide details on below the table presented in this supplement. These signs were collected from one veterinary profession-directed article (Oellig et al., 2024) that posed a direct question on behavioural signs in an animal of its possible historic abuse experience and from two articles on human child signs of abuse experience (Dougherty, 2006, with the original source being: Prevent Child Abuse Georgia. Recognizing physical abuse: http://www.preventchildabusega.org/html/physabuse.html and Nyberg et al., 2022). From the two human child signs of abuse lists, we selected only the signs that regarded visible behaviours, dropping three items from Dougherty (2006) as shown below and six items from Nyberg et al., 2022, with three items grouped under ‘aggression’ as shown below. For the Nyberg et al., 2022 list we first selected the signs scored at higher accuracy (<2.5 mean accuracy score, with the mean ranging from 1.8 to 3.7) as assessed by child professionals after these signs surfacing in a literature review (Nyberg et al., 2022) and the additional indicated signs by the 39 professionals contributing in this study that were also scored at a higher accuracy rating with at least two entries given. We adapted the signs to reflect an animal instead of a child situation, replacing for instance the word ‘child’ for ‘animal’ as shown below.

|  | **Original item** | **Adapted sign description** |
| --- | --- | --- |
| Source: Oellig et al., 2024 |  |  |
|  | Based on an animal’s behaviour, do you think you can identify whether it is being mistreated by its owner? Yes, in most cases, yes, sometimes, no, don’t know/unsure. | *unchanged* |
| Source: Dougherty, 2006 | A child may display the following: | Which of these signs would you regard as possible indicative of an animal’s historic abuse experience? |
|  | Feels deserving of punishment | *dropped item* |
|  | Is wary of adult contact | An animal is wary of adult contact |
|  | Acts apprehensive when others cry | An animal acts apprehensive when a human (adult or child) cries |
|  | Displays behavioural extremes, from aggressiveness to withdrawal | An animal displays behavioural extremes, from aggressiveness to withdrawal |
|  | Seems frightened of parents | An animal seems frightened by owner(s) or of going towards/ with them (going home) |
|  | Is afraid to go home | See above |
|  | Reports injury by parent | *dropped item* |
|  | Displays a vacant of frozen stare | An animal displays a vacant or frozen gaze |
|  | Lies very still while surveying surroundings | An animal sits or lies very still while surveying surroundings |
|  | Responds to questions in monosyllables | *dropped item* |
|  | Has inappropriate or precocious maturity | A young animal shows inappropriate or precocious maturity |
|  | Uses manipulative behaviour to get attention; capable of only superficial relationships | An animal uses demanding (manipulative) behaviour to get attention An animal seems less capable of forming a social bond with humans |
| Source: Nyberg et al., 2022 |  | How would you rate the accuracy of below animal behavioural signs as indicative of an animal’s abuse experience? 7-point Likert-scale (1 = very accurate; 7 = very inaccurate). |
|  | Post-traumatic stress disorder (PTSD) | *dropped item* |
|  | Poor self-esteem | *dropped item* |
|  | Withdrawal | Withdrawal |
|  | Anxiety | Anxiety |
|  | Nightmares | *dropped item* |
|  | Depression | Depression |
|  | Self-harm | Auto mutilation |
|  | Acting out | Aggression |
|  | Emotional problems | *dropped item* |
|  | Inappropriate sexual behaviour | *dropped item* |
|  | Inappropriate anger | Aggression |
|  | Behavioural problems | Behavioural problems |
|  | Aggressive behaviour | Aggression |
|  | Suicidal thoughts | *dropped item* |
|  | Hypervigilant, guarded/insecure behaviour | Hypervigilant, guarded/insecure behaviour |
|  | Anxiety or reluctance to be in the presence of or be left alone with a particular person or to go to a particular place/room, behavioural avoidance | Anxiety or reluctance to be in the presence of or be left alone with a particular person or to go to a particular place/room, behavioural avoidance |
|  | Inappropriate laughter, grimacing or unusual smile, problems with emotion regulation | Acting overly 'pleasing' through for instance submissive behaviour/appeasement |
|  | Expressing fear of parent/other, fear of specific caregiver | Expressing person specific fear |
|  | Change in behaviour without any other explanation, sudden change in behaviour | Sudden behavioural change, which cannot be explained otherwise |
|  | Problems developing friendships with others, lack of social skills | Problems with forming a social bond with humans |
|  | Describing parent/other in ONLY positive ways, (overcompensating*), odd interaction patterns* with parent | Expressing person specific overly 'pleasing' behaviour through for instance submissive behaviour/appeasement |

***Details on the behavioural signs selected from human-child literature sources***

For the human child, behavioural signs of possible historic abuse experience may regard a child being wary of adult contact, coming across as frightened of care providers or of going home, acting apprehensive when others cry, displaying behavioural extremes from aggressiveness to withdrawal, displaying a vacant or frozen gaze, lying very still while surveying surroundings, using manipulative behaviour to get attention and showing lesser ability to form healthy social bonds (Dougherty, 2006).

In another child-directed study, next to for instance physical signs, behavioural signs were mentioned in a third category for child abuse recognition, albeit in the form of ‘behavioural issues’ and grouped with other signs, e.g. regarding disciplining methods (Chia-Jung et al., 2022). In particular, for children with disabilities, behavioural signs may be regarded as highly relevant in abuse recognition as they are at a high risk of suffering abuse and, depending on the challenges these children face, verbal expression of abuse experience may be hampered (Nyberg et al., 2022). Alike the Chia-Jung et al. (2022) study ‘behavioural issues’ are mentioned in literature on this particular population, as behavioural signs possibly indicative of historic abuse experience, in an online survey of 39 child professionals working with children with disabilities and/or child abuse (Nyberg et al., 2022). Whilst acknowledging that other causes may factor in such behavioural issues than abuse related trauma, the study pointed at behavioural issues as a possible way to recognise abuse, through assessing a list of possible abuse signs for their accuracy in abuse indication, using a seven-point Likert-scale ranging from very accurate (‘1’) to very inaccurate (‘7’; Nyberg et al., 2022). The listed signs were derived from a rapid review of 23 included articles and the data extraction tool held fifteen behavioural signs and nineteen physical signs, with the data extraction being challenged by use of inconsistent labels for similar concepts, such as ‘aggression’ and ‘violent behaviour’ (Nyberg et al., 2022). In the accuracy assessment means ranged from 1.8 to 3.7 and of the signs with higher accuracy (<2.5 mean accuracy score) the listed behavioural signs regarded: acting out, aggressive behaviour, anxiety, behavioural problems, depression, emotional problems, inappropriate anger, inappropriate sexual behaviour, nightmares, poor self-esteem, post-traumatic stress disorder (PTSD), self-harm, suicidal thoughts, withdrawal; with physical signs regarding: burns, bruising, penetration signs, or trauma (Nyberg et al., 2022). Twenty of the 39 professionals via free-text comments added abuse signs to the list of literature-derived abuse signs and here a higher accuracy rating with at least two entries was given to behavioural signs of ‘hypervigilant, guarded/insecure behaviour’, ‘anxiety or reluctance to be in the presence of or to be left alone with a particular person or to go to a particular place/room, behavioural avoidance’, ‘inappropriate laughter, grimacing or unusual smile, problems with emotion regulation’, ‘expressing fear of parent/other, fear of specific caregiver’, ‘change in behaviour without any other explanation, sudden change in behaviour’, ‘problems developing friendships with others, lack of social skills’, ‘describing parent/other in *only* positive ways, (overcompensating), odd interaction patterns with parent’ (Nyberg et al., 2022). Thus, although much remains to be studied (Bailhache et al., 2013; Gallione et al., 2017; Saini et al., 2019), for human child-directed abuse, insights on behavioural signs have been gathered and these may possibly serve as a basis to study animals residing under direct human care, such as cats and dogs.

References:

Bailhache M, Leroy V, Pillet P, Salmi LR. Is early detection of abused children possible?: a systematic review of the diagnostic accuracy of the identification of abused children. BMC pediatrics. 2013 Dec 5;13(1):202.

Chia-Jung CH, Yi-Wen CH, Jui-Ying FE. Screening tools for child abuse used by healthcare providers: a systematic review. Journal of Nursing Research. 2022 Feb 1;30(1):e193.

Dougherty K. Recognizing signs of child abuse: Know the clinical guidelines for recognizing child maltreatment. Nursing2020 Critical Care. 2006 Nov 1;1(6):44-9.

Gallione C, Dal Molin A, Cristina FV, Ferns H, Mattioli M, Suardi B. Screening tools for identification of elder abuse: a systematic review. Journal of clinical nursing. 2017 Aug;26(15-16):2154-76.

Nyberg A, Ferm U, Bornman J. Signs of abuse in children with disabilities: A rapid review with expert panel social validation. Journal of Intellectual & Developmental Disability. 2022 Jul 3;47(3):206-17.

Oellig L, Lindsjö J, Röcklinsberg H. Exploring Swedish veterinarians' awareness of non-accidental-injuries, animal abuse and the Link to domestic violence, and their role in addressing this societal issue. Frontiers in Veterinary Science. 2024 Dec 18;11:1439106.

Saini SM, Hoffmann CR, Pantelis C, Everall IP, Bousman CA. Systematic review and critical appraisal of child abuse measurement instruments. Psychiatry research. 2019 Feb 1;272:106-13.
