# Supplementary material for: Abuse recognition by shelter staff and shelter animal adopters
Source: PLoS One. 2026 Mar 11;21(3):e0343066. doi: 10.1371/journal.pone.0343066 (PMC12978493; doi:10.1371/journal.pone.0343066)
Supplement: S3 Table — (DOCX) [file pone.0343066.s003.docx]

**Supplement 3 – Respondent characteristics**

| **Characteristic** | **Percentage of N=23 shelter staff respondents** | **Count of N=23 shelter staff respondents** |
| --- | --- | --- |
| **Estimated yearly number of cats cared for by the shelter were the respondents work** |  |  |
| 0 cats | 4.4% | 1 |
| 1-100 cats | 13.0% | 3 |
| 100-250 cats | 34.8% | 8 |
| 250-500 cats | 39.1% | 9 |
| >500 | 8.7% | 2 |
| **Estimated yearly number of dogs cared for by the shelter were the respondents work** |  |  |
| 0 | 8.7% | 2 |
| 1-50 | 52.2% | 12 |
| 50-100 | 8.7% | 2 |
| 100-200 | 26.1% | 6 |
| >200 | 4.4% | 1 |
| **Profession of shelter staff** |  |  |
| (Mainly) cat caretaker | 34.8% | 8 |
| (Mainly) dog caretaker | 26.1% | 6 |
| (Mainly) management | 26.1% | 6 |
| (Mainly) animal trainer | 8.7% | 2 |
| (Mainly) para-veterinarian | 4.4% | 1 |
| **Year of experience in present profession** |  |  |
| 0-2 years | 39.1% | 9 |
| 2-5 years | 21.7% | 5 |
| 5-10 years | 17.4% | 4 |
| >10 years | 21.7% | 5 |
| **Gender of shelter staff** |  |  |
| Female | 78.3% | 18 |
| Male | 17.4% | 4 |
| Other/prefer not to say | 4.3% | 1 |
| **Age of shelter staff** |  |  |
| 18-35 years | 30.4% | 7 |
| 25-50 years | 30.4% | 7 |
| 50-65 years | 30.4% | 7 |
| >65 years | 8.7% | 2 |

| **Characteristic** | **Percentage of N=132 shelter animal adopter respondents** | **Count of N=132 shelter animal adopter respondents** |
| --- | --- | --- |
| **Animal for which they fill out survey** |  |  |
| Cat | 30.3% | 40 |
| Dog | 69.7% | 92 |
| **Origin of adopted animal** |  |  |
| Shelter abroad | 37.9% | 50 |
| Shelter abroad via shelter in The Netherlands | 5.3% | 7 |
| Shelter in The Netherlands | 38.6% | 51 |
| Other (e.g. via organisation rehoming from home to home) | 18.2% | 24 |
| **Age of animal at adoption** |  |  |
| <0.5 year | 13.6% | 18 |
| 0.5-1 year | 20.4% | 27 |
| 1-3 year | 34.9% | 46 |
| >3 years | 31.1% | 41 |
| **Years of animal in care** |  |  |
| <2 months | 3.0% | 4 |
| 2-6 months | 3.0% | 4 |
| 6 months-1 year | 10.6% | 14 |
| 1-3 years | 31.1% | 41 |
| >3 years | 52.3% | 69 |
| **Reason animal was up for adoption as presented to the owner** |  |  |
| Life event | 18.9% | 25 |
| Behavioural issue of aggression | 9.1% | 12 |
| Behavioural issue of fear | 11.4% | 15 |
| Behavioural issue - other than aggression or fear | 10.6% | 14 |
| Neglect | 46.2% | 61 |
| Physical abuse | 11.4% | 15 |
| **Information provided by rehoming organisation on the animal’s historic abuse** |  |  |
| Yes, with certainty | 6.1% | 8 |
| Yes, likely | 15.2% | 20 |
| No, not known | 67.4% | 89 |
| Unsure | 11.4% | 15 |
| **Animal adopter thinks animal was abused before coming to live with them** |  |  |
| Yes, with certainty | 38.6% | 51 |
| Yes, likely | 36.4% | 48 |
| No | 25.0% | 33 |
| **When mistreated, how does the animal adopter think the animal was mistreated (N=182 times reported, so some animals multiple forms)** |  |  |
| Neglect | 26.9% | 49 |
| Abuse – mental | 36.3% | 66 |
| Abuse – physical | 36.8% | 67 |
| **Gender of shelter animal adopters** |  |  |
| Female | 91.7%% | 121 |
| Male | 6.1% | 8 |
| Other/prefer not to say | 2.3% | 3 |
| **Age of shelter animal adopters** |  |  |
| 18-35 years | 13.6% | 18 |
| 25-50 years | 26.5% | 35 |
| 50-65 years | 44.7% | 59 |
| >65 years | 14.4% | 19 |
| Prefer not to say | 0.8% | 1 |
