# Supplementary material for: Abuse recognition by shelter staff and shelter animal adopters
Source: PLoS One. 2026 Mar 11;21(3):e0343066. doi: 10.1371/journal.pone.0343066 (PMC12978493; doi:10.1371/journal.pone.0343066)
Supplement: Attachment — Submitted filename: Response to Reviewers.pdf [file pone.0343066.s005.pdf]

Utrecht, November 5th, 2025

Dear Academic Editor and Reviewers,

Thank you for your response that allows us to improve our manuscript titled ‘Abuse recognition by shelter staff and shelter animal adopters’. We have carefully considered the provided feedback. Please find here below, our response to each point raised. Please note that line indications refer to the manuscript without track changes, for your easy reading.

Sincerely,

Ineke R. van Herwijnen Ph.D.

i.r.vanherwijnen@uu.nl

Division of Animals in Science and Society, Faculty of Veterinary Medicine, Department of Population Health Sciences, Utrecht University, Utrecht, The Netherlands

Response to each point raised:

Editor notes that require a response

2. Please provide additional details regarding participant consent. In the ethics statement in the Methods and online submission information, please ensure that you have specified what type you obtained (for instance, written or verbal, and if verbal, how it was documented and witnessed). If your study included minors, state whether you obtained consent from parents or guardians. If the need for consent was waived by the ethics committee, please include this information.

We have adapted this section and copied information that was previously only in the section on respondent recruitment. Changes are indicated in lines 99 to 103.

3. When completing the data availability statement of the submission form, you indicated that you will make your data available on acceptance.

The data file is uploaded in the system.

4. Please amend either the title on the online submission form (via Edit Submission) or the title in the manuscript so that they are identical.

The short title is now in the online submission form.

Additional Editor Comments: I strongly encourage you to thoroughly check the grammar and spelling of your manuscript before resubmission.

Thank you for pointing this out, the manuscript was thoroughly checked and adapted throughout.

Reviewer's Responses to Questions:

Reviewer #1: This study provides original insights into a topic that has remained significantly underexamined in recent years. Its findings are expected to make a meaningful contribution to the academic discourse surrounding the detection of animal abuse. Nonetheless, revisions should be implemented to enhance the clarity of terminology and to more precisely delineate the study’s limitations.

Thank you for your kind words and suggestions for improving the manuscript.

Introduction: It is unclear why the authors assert that intentional trauma infliction is not always clearly distinguished from the more passive animal neglect. For this academic paper is fundamental the definition of emotional abuse but the authors only offer an example, please define it.

Thank you for pointing out this opportunity for improving our manuscript. We have rewritten the introduction, based on other feedback. We have provided more focus by providing details on physical animal abuse only. In line with this focus, we provide a definition in lines 59 to 61: ''active, physical, with serious violence or direct harm to animals including fractures induced by kicking, injuries induced by falling, burning, or performing home surgery'. Please note that we also indicate that multiple definitions exist.

It is mentioned that the veterinary clinical setting may come with challenges for assessing animal behavioral signs, highlighting shelters as an alternative. However, it does not elaborate on why shelters represent a preferable option.

We have added this elaboration in lines 80 to 82, as following; 'Shelters may harbour animals that were previously abused, such as when seized, which can be on different grounds, including abuse [37]. After acclimatisation to a shelter surrounding, a broader range of behaviours in animals may be seen than in the veterinary clinical setting [38]'.

Methods: Although signs of emotional abuse in children may help guide the identification of behavioral indicators in animals, it is essential to clarify the criteria used to determine which aspects of children's behavior are suitably applicable to animals.

We now describe the selection approach using the specific word 'criterium' in lines 129 to 132, with as the main text: 'As our study regarded a first step towards behavioural signs of animal abuse only, we aimed to include as many of the behavioural signs known from child abuse to assess how our study samples would rate their likely application to animal abuse situations. Thus, our only exclusion criterium was a listed sign not regarding visible behaviour, such as 'feels deserving of punishment'.

Based on the reviewer's feedback we also, additionally, stress the following. We do not regard the output of this study suitable behavioural signs for studying animal abuse. Assessing such suitability will require further studying, using different study methods than applied in this first step. Lines 379 to 391 in the discussion section: 'Our study is a first step towards identifying behavioural signs of animal abuse. Our study findings cannot be used to assess animal abuse based on behavioural signs. The findings are a first step towards a future possibility for such assessment. By establishing deemed relevance, we have not yet established deemed value of behavioural signs in animal abuse assessment. Behaviours of interest need to be studied further in observational studies of animals, preferably comparing the behaviour of animals that have suffered animal abuse to that of animals that have not suffered animal abuse. Once behavioural differences have objectively been established the behaviours will next need to be validated for their potential use to indicate animal abuse. It is likely that in these studies seized sheltered animals will be studied. Their caretakers may be asked to report on animal behaviour, including on possible behavioural signs of animal abuse. Our study highlights that shelter animal staff and adopters may differ in their recognition of behaviours other than fear, when assessing likeliness of animal abuse. Consequently, either training caretakers involved in animal behaviour studies in their reporting of animal abuse, and/ or using additional validation sources, may benefit the quality of studies on animal abuse'

Discussion: The difficulty in detecting behavioral indicators of animal abuse is acknowledged; therefore, it is important to clearly outline the specific challenges involved in identifying abuse based on behavioral signs. Finally, given that the study's results are based on the perceptions of non-experts, the limitations should clearly state that the findings do not determine the primary behavioral indicators for suspecting animal abuse.

Thank you for addressing that these points could have been made more specifically in our manuscript. The text indicated in lines 379 to 391 and specified above, makes this more specific in the new version of our manuscript.

Reviewer #2: 1) The terms used within this research are often vague or undefined. For instance, "aggression" is a multifaceted set of distinctly identifiable behaviors, many of which have additional significance based on context. This needs to be defined. "Seems wary of adult contact", "Seems less capable of forming human bonds", etc. are amorphous judgement calls not defined to the survey respondents. These characteristics are presumably also occurring in a shelter environment, which is stressful and has been clearly identified as unlikely to produce reliable, reproducable results. "Precocious maturity" in a domestic dog is an inappropriate measure - puppies do not show such behavior. Other factors in Supporting #2 are inappropriately projected from human children to dogs or have potentially ery different emotional or behavioral bases. Dogs are not humans.

Thank you for your feedback and we could not agree more! This is also why we addressed in our discussion section the need for establishing an ethogram. Based on your feedback we have at several points in our manuscript highlighted more how this study is merely a first step in studying the indication of behavioural signs of animal abuse. Also, we have added in lines 379 to 391 a section which may make the limitations of our study clearer: 'Our study is a first step towards identifying behavioural signs of animal abuse. Our study findings cannot be used to assess animal abuse based on behavioural signs. The findings are a first step towards a future possibility for such assessment. By establishing deemed relevance, we have not yet established deemed value of behavioural signs in animal abuse assessment. Behaviours of interest need to be studied further in observational studies of animals, preferably comparing the behaviour of animals that have suffered animal abuse to that of animals that have not suffered animal abuse. Once behavioural differences have objectively been established the behaviours will next need to be validated for their potential use to indicate animal abuse. It is likely that in these studies seized sheltered animals will be studied. Their caretakers may be asked to report on animal behaviour, including on possible behavioural signs of animal abuse. Our study highlights that shelter animal staff and adopters may differ in their recognition of behaviours other than fear, when assessing likeliness of animal abuse. Consequently, either training caretakers involved in animal behaviour studies in their reporting of animal abuse, and/ or using additional validation sources, may benefit the quality of studies on animal abuse.'

In addition, we have now not only indicated in the methods section that our study followed the methodology of two scientific studies on child abuse – using their terminology as a starting point. In addition to this section and the supplement with further details, we have now also added these lines 358 to 367 in the discussion section: '(…). Also, we deemed it necessary for this first step to stay close to the approach taken in the child abuse literature that formed the basis of this study. Thus, we did not work with detailed descriptions of behavioural signs. Although these signs could form a basis for future studies, we point at the need to further specify behaviours in an ethogram. To date, when animal abuse literature describes behavioural signs, these are often described in general terminology, without behavioural specifications, such as 'signs of distress', 'the animal displays an unexplained change in behaviour', or 'observations of normal/abnormal behaviour' [6]. Establishing an ethogram that includes behaviours that objectively and discriminatively describe those behaviours that may be of interest as behavioural signs of animal abuse is one of the many steps for future studies.'

2) Sample size is quite small as identified by the authors.

We have further highlighted this in the discussion section in line 358: 'Our study's limitations are not only in the limited number of participants in each sample.' Note that despite substantial rewriting of the abstract, we have kept the sample sizes as to make the small-scale of the study clear to our readers at first glance.

3) The claims of actual abuse are poorly documented. Abuse is variously interpreted by adopters and shelter staff without clear definition. Were the police referrals prosecuted? Successfully? Were the police cases assumed to be valid without prosecution or conviction?

We have reworded this text in lines 167 to 168: 'We also asked if shelters *generally* gathered or received information on animals in their care suffering animal abuse.'

4) Sweeping conclusions are made that may not be supported by confirmed research: i.e. "Person-specific fear behavior was regarded a more accurate indicator of animal abuse than aggression, problems with human-social bond formation and person-specific pleasing behavior." Abstract line 43-46. According to whom? 'Aggression', 'person-specific fear', and 'problems with human-social bond formation' are undefined and unsupported.

Thank you for indicating how we could improve our abstract. We have now reworded sections to adhere to your feedback. E.g. in lines 35 to 36: 'Therefore, we aimed to study if behavioural signs from studies on child abuse are viewed as relevant by shelter staff and shelter animal adopters, (…)' and in lines 40 to 41: 'Person-specific fear was indicated by shelter staff and shelter animal adopters as a more accurate indicator of (…)'.

5) The expression of factors suffers from the presumably Dutch to English translation. Wording is awkward and convoluted, causing potential or real misunderstanding. I.e. "Environmental signs may regard an animal caretaker's higher alcohol consumption or a weaker belief in justice." Introduction, line 86. What? What signs? How much alcohol? Compared to whom? "This, as animals may show different suppressed, and/or fear behaviors due to fear of veterinary surroundings and previous painful experiences in the veterinary clinical setting." I understand the idea, but the wording is convoluted and may not be clear to others. Also see line 247 "...being frightened of the owner or going towards them...". If you can't go toward or away, then what can the animal do?

Thank you for highlighting the sections with convoluted sentences. The manuscript was thoroughly checked and adapted throughout.
