# Supplementary material for: Abuse recognition by shelter staff and shelter animal adopters
Source: PLoS One. 2026 Mar 11;21(3):e0343066. doi: 10.1371/journal.pone.0343066 (PMC12978493; doi:10.1371/journal.pone.0343066)
Supplement: Attachment — Submitted filename: Response to Reviewers.docx [file pone.0343066.s006.docx]

Utrecht, January 8^th^, 2026

Dear Academic Editor and Reviewers,

Thank you for the second review of our manuscript titled ‘Abuse recognition by shelter staff and shelter animal adopters’. We have carefully considered the provided feedback. Please find below our response to the points that were raised. Please note that line indications refer to the manuscript without track changes, for your easy reading.

Sincerely,

Ineke R. van Herwijnen Ph.D.

i.r.vanherwijnen@uu.nl

Division of Animals in Science and Society, Faculty of Veterinary Medicine, Department of Population Health Sciences, Utrecht University, Utrecht, The Netherlands

***Response to each point raised:***

***Reviewer's feedback:***

*Reviewer #1:*

Reviewer #1: Research on emotional abuse in animals is essential for detecting and prosecuting cases of animal cruelty. The authors have strengthened the manuscript and successfully addressed all of my comments.

**Thank you again for your review and valuable time.**

*Reviewer #3:*

**Thank you for your feedback and we now have more clearly brought forward two things. Firstly, we write more on our rationale for studying the two populations from which our samples originated. Secondly, we bring forward more strongly how we deem this study a small first step towards other studies that are of higher scientific strength, such as studies objectively observing animal behaviours, based on an ethogram and done so by trained observers. We feel that our present small-scale study will help to point at the importance of such studies and initiate such studies, which might be deemed challenging to set up and execute. Below we provide more details on the changes to the manuscript.**

1. The participants in the study, shelter workers with various amounts of training and experience with animals in general and abused animals in particular, and shelter adopters, who likely have even less training or experience with animals, are surveyed for their opinions. These non-experts may or may not represent what the people on the "front lines" of shelters may believe are the behavioral signs of animal abuse, but if the goal of this research is to point towards an ethogram, then we need to look not at the opinions of non-experts, but in the actual behaviors of actual animals known to have been abused. In short, it would be most appropriate to determine which behaviors can reliably be correlated to animal abuse and then determine if those behavior are being appropriately considered by the staff and adopters who interacted with sheltered animals. Starting with the views of non-experts of various backgrounds only serves to lead research off course in this area.

**Thank you for highlighting a concern that this first step should not lead to inclusion or exclusion of possible behaviours in an ethogram for studying the likeliness of an animal’s possible historic abuse experience. We have rephrased sentences to ensure this is not unintentionally suggested and that we merely intend to point at the importance of such ethogram-based studies and the need to initiate such studies. E.g. in line 401 onwards: ‘Our study is a first step towards identifying behavioural signs of animal abuse. Our study findings cannot be used to assess animal abuse based on behavioural signs. We stress that the surveying of our particular study populations of shelter animal staff and adopters, comes with a limited use of our results. When constituting an ethogram of behaviours that may serve as possible abuse signs, our present study should not be used to determine the inclusion or exclusion of possible behaviours. We did not work with trained observers and did not study the animals themselves. Value of our study is in having gathered a first insight into our sample’s views on possible abuse signs. The findings indicate the relevance of a future possibility for such assessment. By establishing deemed relevance, we have not yet established deemed value of behavioural signs in animal abuse assessment. Behaviours of interest need to be identified and studied in observational studies of animals, preferably comparing the behaviour of animals that have suffered animal abuse to that of animals that have not suffered animal abuse. Once behavioural differences have objectively been established the behaviours will next need to be validated for their potential use to indicate animal abuse.’**

**We have more clearly indicated why we opted for shelter staff and shelter animal adopters as study populations, from line 94 onwards: ‘We have two reasons for involving shelter staff and animal adopters. The first reason is that abused animals form a part of the animal population under their care, as indicated in the previous paragraph [37]. This makes for an accessible population that may have under their care the animals of interest. Other such populations, are difficult to reach, due to it for example being unsafe or unethical to survey domestic abuse victims who have or had animals under their care, or to study animals seized, but not released and possibly involved in a legal trajectory. Also, deceased animals cannot be studied for behaviour, which is a contrast to studies assessing physical abuse signs. The second reason is that shelter staff and adopters may have views on possible previous abuse of animals under their care. These views are of interest as presently little is known on the validity of such views in regard to appointing an animal’s previous abuse or lack thereof. Learning more about these views can help us to not wrongfully appoint or reject a history of animal abuse.’**

2. On a related note, I question the reliance on the behavioral signs of child abuse as the basis for this study. For example, person-specific fear in a pet cat or dog may be a sign of physical abuse; however, it may also be a sign that the pet was not adequately socialized during their short but critical socialization window (feline - 2-8 weeks, canine 3-14 weeks) to a particular demographic of humans, as is frequently the case with animals taken from feral colonies, animals raised by inexperienced (not not abusive) breeders, and so on. Thus, while this sign may be quite reliable in children, it may be less so in pets whose life experiences can vary greatly from children. Further, the original source on child abuse was a continuing education article for nursing professionals (33). In short, we should not anthropomorphize animals.

**We feel there is relevance in studying behavioural signs of child abuse for their possible relevance in animals. Humans are known to show comparisons (though not similarity) in how they approach children and cats/dogs. E.g. attachment bonds and parenting styles apply to the interhuman relationships as well as to the relations between humans and cats/dogs to a degree^1-6^. We agree that we should not anthropomorphize animals. To prevent anthropomorphism, information is necessary on whether people, such as shelter animal staff and adopters, may or may not be inclined to do so. We do not feel that our study should be an end point. Contrary: we agree that other methods are highly necessary to assess which behavioural signs may or may not be relevant tot indicating an animal’s likely historic abuse, or lack thereof. We have strengthened the information on that point, as indicated under point 1) above. Additionally, we suggest future studies to consider training animal caretakers if these would be questioned on animal abuse behavioural signs (line 413 onwards): ‘(…). It is likely that in these studies seized sheltered animals will be studied. Their caretakers may be asked to report on animal behaviour, including on possible behavioural signs of animal abuse. Our study highlights that shelter animal staff and adopters may differ in their recognition of behaviours other than fear, when assessing likeliness of animal abuse. Consequently, either training caretakers involved in animal behaviour studies in their reporting of animal abuse, and/ or using additional validation sources, may benefit the quality of studies on animal abuse.’**

**1. Brubaker L, Udell MA. Does Pet Parenting Style predict the social and problem-solving behavior of pet dogs (Canis lupus familiaris)?. Animal cognition. 2023 Jan;26(1):345-56.**

**2. Kuo CH, Kessler S. Intergenerational transmission of human parenting styles to human–dog relationships. Animals. 2024 Mar 28;14(7):1038.**

**3. Luchesi SH, Machado DS, Trindade PH, Mikulincer M, Otta E. Psychometric validation of the Brazilian Version of the Pet Attachment Questionnaire (PAQ): An examination of predictors of attachment styles among cat owners. Applied Animal Behaviour Science. 2022 Nov 1;256:105769.**

**4. Rehn T, Beetz A, Keeling LJ. Links between an owner’s adult attachment style and the support-seeking behavior of their dog. Frontiers in psychology. 2017 Nov 30;8:2059.**

**5. Stoeckel LE, Palley LS, Gollub RL, Niemi SM, Evins AE. Patterns of brain activation when mothers view their own child and dog: An fMRI study. PloS one. 2014 Oct 3;9(10):e107205.**

**6. Volsche S, Gray P. “Dog moms” use authoritative parenting styles. Human-animal interaction bulletin. 2016 Dec 1(2016).**

3. The sample sizes were very small for this study, and there is a strong likelihood of voluntary response bias given the topic and the non-random manner in which participants were recruited. The results cannot be deemed to represent shelter workers or shelter adopters in general.

**We agree and have strengthened text sections addressing this limitation of our study, such as in the abstract in line 49-51: ‘Studying such differences in larger samples may provide insight into how these populations assess an animal’s possible abuse experience or lack thereof.’ and the discussion section, line 376 onwards: ‘Due to this limited number of participants, we caution against extrapolation of our findings to the total (Dutch) population of shelter staff and animal adopters.’. Also, we deemed in necessary from the start to stress the low numbers, such as in the abstract in line 39.**

4. Many of the survey items reference vague descriptions that should have been more precisely defined. For example, terms such as "aggression,' "wary," and "demanding behavior," to name just a few, are not clear and could have many interpretations.

**Thank you for allowing us to explain that we opted to stay close to the original wording used in the child abuse studies that formed the basis of this study. We do not see our study as having the scientific strength that an objective animal behaviour observation study would have. We argument at several points in our manuscript the necessity of this studying. However, we feel that our study increases the chance that such studies will be set up and executed. We have strengthened the section in the discussion on this particular limitation of our study, in line 378 onwards: ‘Also, we deemed it necessary to stay close to the approach taken in the child abuse studies that formed the basis of our study. This allows for the child abuse study outcomes to be compared to the outcomes of this study. Consequently, we opted not to work with objective descriptions of animal behavioural signs. Thus, there is a need for such objectively defined behavioural descriptions, specified in an ethogram, to form the basis of an objective assessment of animal abuse signs in future studies. To date, when animal abuse literature describes behavioural signs, these are often described in general terminology, without behavioural specifications, such as ‘signs of distress’, ‘the animal displays an unexplained change in behaviour’, or ‘observations of normal/ abnormal behaviour’ [6]. Establishing an ethogram that includes behaviours that objectively and discriminatively describe those behaviours that may be of interest as behavioural signs of animal abuse is one of the many steps to be taken in future studies.’.**

5. Considering signs of physical abuse in a shelter environment is exceptionally challenging, as the environment itself is a confounding factor in an animal's behavior. While, ultimately, shelters may be places where many abuse cases might be discovered, it would be more useful to study known abuse cases in home or foster settings where other confounding stressors might be better accounted for.

**Thank you for your interesting suggestion to set up a study with these particular study setting characteristics. We agree that shelter settings and shelter animal adoptions come with challenges and this underpinned our second reason to aim at gaining insight into e.g. shelter staff views. This may aid to take such factors into account in future, when assessing animal abuse. We discuss how people’s views (that is in our study the views of shelter staff and shelter animal adopters) may factor in as confounders, e.g. in line 355 onwards: ‘Additionally, expectations of why animals are shelter-surrendered may affect shelter staff and animal adopters differently. We know of no studies comparing such expectations between these two groups, but some studies into shelter animal adopters’ experiences are available. These studies indicate prior concerns of those contemplating to adopt a cat or dog, such as regarding an unknown history of the animal or their unwanted behaviours [47]. Such concerns may be valid, as 72% of N=192 adopted dogs reportedly showed undesired behaviour in the first week after adoption, such as aggression (24%) and fearfulness (21%) [37]. Undesired behaviour was reported at higher rates for dogs that suffered ‘abuse’ (no precise definition for abuse was provided in this study; [37]). After half a year the rates of reported behaviour were lower for fear, but not for aggression [37], and aggression was listed as a return reason for adopted cats and dogs [48]. Often provided reasons to opt for adoption when contemplating to acquire a cat or dog are for it to be ethically or morally correct [49]. So, when a choice for a shelter adoption is made ‘to do good’ and the animal shows undesired behaviours, expectations of a rescued animal ‘being grateful’ and ‘good’ may be unfulfilled. Consequently, it may be helpful to explain such unfulfilled expectations through a previous owner mistreating the animal and behavioural signs may more readily be viewed as a sign of animal abuse. (…)’.**
