# Supplementary material for: Abuse recognition by shelter staff and shelter animal adopters
Source: PLoS One. 2026 Mar 11;21(3):e0343066. doi: 10.1371/journal.pone.0343066 (PMC12978493; doi:10.1371/journal.pone.0343066)
Supplement: Attachment — Submitted filename: Response_to_Reviewers_auresp_3.docx [file pone.0343066.s007.docx]

Utrecht, January 24^th^, 2026

Dear Academic Editor and Reviewers,

Thank you for the new review of our manuscript titled ‘Abuse recognition by shelter staff and shelter animal adopters’. Please find below our response to the points that were raised.

Sincerely,

Ineke R. van Herwijnen Ph.D.

i.r.vanherwijnen@uu.nl

Division of Animals in Science and Society, Faculty of Veterinary Medicine, Department of Population Health Sciences, Utrecht University, Utrecht, The Netherlands

***Response to each point raised:***

**Thank you for addressing the comments from the previous reviewers. At this point, there are a few areas of the manuscript that could benefit from grammatical checks and rewording, particularly in the introduction. Additionally, the introduction requires clarification, which will assist in addressing the reviewer's comments regarding study limitations. Below, you will find my suggestions. I also recommend consulting a program or service to assist with these grammatical changes. Once these revisions are made, I will review the manuscript again and proceed to accept it for publication.**
Thank you and we have made grammatical changes, particularly in the introduction section.

**Line 57: Please rephrase, as the current wording is confusing. Perhaps something along the lines of..."****Artificial selection has resulted in various traits that can sometimes negatively impact the overall well-being of domesticated species".**
The text was adapted.

**Lines 60-61: If you argue that animal abuse exists in our “societies”, please support your claim with data from multiple countries.**
Thank you for this suggestion, we added references from multiple countries.

**Lines 61-63: Are you suggesting that most people view animal abuse negatively and consider animals to be the victims in these situations?**
We have replaced the word ‘victims’.

**Lines 63-64: It's difficult to determine whether your study is specifically focused on physical abuse, as you are only surveying shelter workers and prospective adopters about their general perceptions of whether the animals have been abused in the past. Additionally, you're asking which signs they believe to be the most reliable indicators of abuse. For this reason, you should consider addressing all aspects of animal abuse in your review.**
We are specifically interested in perceptions of physical abuse and have stated this at several point in the manuscript, also explaining why we have this study interest. Previous review led to removal of information on alternative forms of animal abuse, such as emotional animal abuse, as to not confuse readers and avoid unintentionally indicating a broader scope of our study.

**Lines 67-68: I believe this can be combined with the previous sentence for clarity. “Various definitions of animal abuse exist; however, in our current study, we define it as...”**
We have made this adaptation.

**Line 78-79: This undermines your study because it focuses solely on the behaviors that shelter workers and staff associate with abuse. Additionally, based on the citations provided, there has been some research conducted on this topic. I recommend omitting the current content and summarizing what is known about behavioral indicators so far, even if the information is limited.**
We have made changes that should more clearly communicate what we aimed to state with this section.

**Lines 81-89: I would rephrase this paragraph to emphasize that it is easier to distinguish behavioral indicators of abuse in shelter settings compared to other contexts. Right now, the wording is unclear.**
We have adapted this text section. Unfortunately, we cannot yet say that it is easier to distinguish behavioural indicators of abuse in shelter settings compared to other contexts – such comparative studies are to our knowledge non-existent. We agree with one of the previous reviewers who pointed this out.

**Line 87-89: To be cautious, I would recommend softening this language a bit, especially since there may be data reports available that are not published in scientific journals. This highlights a common issue in research on domesticated animal behavior, which could be worth mentioning.**
We have reworded these lines.

**Lines 91-104: Similar to my previous comment, please clarify that y****our initial focus is on assessing whether signs of animal abuse are perceived as significant by staff members and potential adopters. In the second step, you will evaluate which signs they consider to be the most prominent. Many studies compare the accuracy of veterinarians' perceptions with those of non-veterinarians regarding animal behavior. These studies should be cited to support the idea that experience results in higher accuracy.**
We have added the information on the steps, while trying to maintain the text requested by a previous reviewer. Adding literature on veterinarian’s perceptions, would come with a risk of wrongfully suggesting that our present study, with a small number of participants only, can already be used for purposes such as assessing an effect of experience level in shelter staff.

**Lines 92-93: You need to better explain the rationale behind linking child abuse to animal abuse in the current study. Additionally, the information from lines 74-79 could be integrated here, as it currently seems out of place.**
We added this text as to adhere to a previous reviewer’s comments, but have made adaptations in both text sections to improve the flow in the introduction.

**Line 96: Remove “as indicated in the previous paragraph”. Please combine the sentences from lines 95-96 with the sentence from lines 96-97 for clarity.**
This text section has been removed.

**Line 106: Just state “Our current study will focus on domesticated dogs and cats”.**
This sentence was adapted.

**Lines 106-112: Clearly specify how the behavioral indicators of fear manifest. Additionally, for "other" behaviors, aim to be more specific (e.g., prosocial behaviors, aggressive behaviors, etc.). Consult lines 309-313, as it appears you included this information in the discussion but not the introduction.**
This text section was extended.

**Lines 122-124: Please place this before the previous paragraph.**
This section was replaced to the start of the Ethics section.

**Lines 320-321: Remove this sentence.**
We have removed the sentence: ‘As such, attachment/ attention-seeking was of interest to us.’

**Lines 340-342: Reword for clarity.**

Thank you, we have rewritten this section.

**Lines 401-402: Please reword it as follows: “Our study represents an initial step in identifying behavioral signs of abuse that are noticeable to human observers. However, it is important to clarify that our research does not evaluate the accuracy of human assumptions in relation to the actual experiences of the animal”. You need to accurately portray your study, which focuses on human perceptions. Also, please add this caveat to your introduction.**

We have replaced this sentence and added the information at the end of the Introduction section also.
